# Puerperal women’s satisfaction with the obstetric services received: improvement of an assessment instrument

**DOI:** 10.1590/0034-7167-2022-0457

**Published:** 2023-11-27

**Authors:** Lourivaldo Bispo Alves, Cristiane Faiad, Carlos Manoel Lopes Rodrigues, Ângela Ferreira Barros

**Affiliations:** I Fundação de Ensino e Pesquisa em Ciências da Saúde. Brasília, Distrito Federal, Brazil; II Universidade de Brasília. Brasília, Distrito Federal, Brazil; III Centro de Ensino Unificado de Brasília. Brasília, Distrito Federal, Brazil

**Keywords:** Women’s Health, Parturition, Patient Satisfaction, Surveys and Questionnaires, Health Services Research, Salud de la Mujer, Parto, Satisfacción del Paciente, Encuestas y Cuestionarios, Investigación sobre Servicios de Salud, Saúde da Mulher, Parto, Satisfação do Paciente, Questionários, Avaliação de Serviços de Saúde

## Abstract

**Objectives:**

to improve an instrument that measures postpartum women’s satisfaction with obstetric care.

**Methods:**

action research, developed from a preliminary version of an instrument prepared by nurse-midwives working in public services in the Federal District. The analysis of the results of application of instrument carried out in a pilot test, analysis of evidence of instrument validity, literature review, focus group with the instrument’s developers and interview with the target audience were carried out.

**Results:**

factorial analysis showed three existing factors in the construct. Seven nurses participated, discussing the instrument reformulation, and 20 mothers reported their perceptions about the care received during childbirth, generating five thematic units.

**Final Considerations:**

instrument improvement occurred through item and response scale reconstruction and reorganization, in addition to application of a pre-test with the target population, resulting in an instrument composed of 13 items.

## RESUMO

**Objetivos:**

aprimorar um instrumento que mensura a satisfação de puérperas com a assistência obstétrica.

**Métodos:**

pesquisa-ação, desenvolvida a partir de uma versão preliminar de um instrumento elaborado por enfermeiras obstetras atuantes em serviços públicos do Distrito Federal. Executou-se a análise dos resultados da aplicação do instrumento realizada em um teste piloto, análise de evidência de validade do instrumento, revisão de literatura, grupo focal com as elaboradoras do instrumento e entrevista com o público-alvo.

**Resultados:**

a análise fatorial mostrou três fatores existentes no construto. Sete enfermeiras participaram, discutindo a reformulação do instrumento, e 20 puérperas relataram suas percepções sobre a assistência recebida durante o parto, gerando cinco unidades temáticas.

**Considerações Finais:**

o aprimoramento do instrumento ocorreu por meio da reconstrução e reorganização dos itens e da escala de respostas, além da aplicação de um pré-teste com a população-alvo, resultando em um instrumento composto por de 13 itens.

## RESUMEN

**Objetivos:**

mejorar un instrumento que mide la satisfacción de las puérperas con la atención obstétrica.

**Métodos:**

investigación acción, desarrollada a partir de una versión preliminar de un instrumento elaborado por enfermeros obstetras que trabajan en los servicios públicos del Distrito Federal. Se realizó el análisis de los resultados de la aplicación del instrumento realizado en una prueba piloto, análisis de evidencias de validez del instrumento, revisión de literatura, grupo focal con los desarrolladores del instrumento y entrevista al público objetivo.

**Resultados:**

el análisis factorial mostró tres factores existentes en el constructo. Participaron siete enfermeros, discutiendo la reformulación del instrumento, y 20 madres relataron sus percepciones sobre la asistencia recibida durante el parto, generando cinco unidades temáticas.

**Consideraciones Finales:**

la mejora del instrumento se dio a través de la reconstrucción y reorganización de los ítems y de la escala de respuesta, además de la aplicación de una preprueba con la población objetivo, resultando un instrumento compuesto por 13 ítems.

## INTRODUCTION

User satisfaction has been understood as a quality indicator, based on the perception of the care received, on their expectations and on previous experience with other services^(1-2)^. It is an important tool in assessing the quality of care, which also allows participation and defense of rights in public health services. In the context of the Brazilian Health System (SUS - *Sistema Único de Saúde*), it is one of the mechanisms used in planning, allowing assessing the efficiency and effectiveness of health actions^(3)^.

In the consultations carried out in the SUS, women are the most prevalent^(4)^. In this population group, childbirth assistance can be highlighted, which occurs mostly in hospitals^(5)^, moment in which pregnant women seek the health service to experience childbirth and birth, a process that impacts their lives and that of their families, linked not only to biological aspects, but also to social and psychological ones, which must consider their previous experiences^(6)^.

The positive experience of childbirth is a moment when the parturient meets or exceeds her previous personal and sociocultural expectations, including, in this context, satisfaction with the care received during the process^(7)^. The experience of childbirth, when it is negative, can have serious consequences and damage to maternal and child health immediately or in the long term^(8-9)^.

In this regard, it is based on understanding the level of satisfaction of puerperal women that it is possible to guide the actions that must be developed. After all, by assessing the quality of care for women, it is possible to manage obstetric services and develop strategies to implement public policies for comprehensive care for women’s health^(10-13)^.

Therefore, in order to know postpartum women’s satisfaction in relation to the care received in obstetric services in the Federal District, the Technical Chamber of Obstetric Nursing led the development of a preliminary measure to assess postpartum women’s satisfaction in 2019. This technical chamber is made up of 13 nurse-midwives belonging to the effective staff of the State Department of Health of the Federal District (SES-FD). A collegiate instance is constituted, of an advisory and propositional nature, technically linked to the Board of Nursing, with the attribution of providing advice to the board and its managements, carrying out various activities aimed at improving the work processes of public services for women’s health care in the Federal District (FD).

This preliminary instrument began to be applied as a pilot test in a public hospital of SES-FD and, from that, the importance of making adjustments before standardizing it in other obstetric services was perceived. Based on this demand, the researchers proposed to act collaboratively to improve the instrument. It is believed that, acting in this way, from instrument use in the preliminary version and ensuring the participation of these nurses in this process, the instrument could be more valued and with a greater chance of being implemented in all SES-FD services, projecting greater social insertion of the product of this research.

## OBJECTIVES

To improve an instrument for assessing puerperal women’s satisfaction with obstetric care services.

## METHOD

### Ethical aspects

The research was approved by the Research Ethics Committee of FD’s Foundation for Teaching and Research in Health Sciences. Ethical and legal aspects were respected at all stages of the research, in accordance with Resolution 466/12 of the Brazilian National Health Council.

### Study design

This is action research, with a qualitative and quantitative approach. Action-research allows the interaction of researchers and subjects involved in a cooperative way, in which everyone seeks solutions to the problems experienced, associating theory and practice in the search for reality transformation^(14-15)^.

### Methodological procedures

#### Study setting

This study was carried out in a rooming-in (RI) room at a public hospital in the FD (8^th^ phase).

#### Study participants

Seven nurse-midwives, members of the Technical Chamber of Obstetric Nursing (6^th^ phase), and 20 postpartum women admitted to RI (8^th^ phase) participated in the study.

#### Data collection, organization and analysis

The study was developed between September 2019 and October 2021, and used the twelve phases of the action research structured by Thiollent^(14)^ as a guide, as described below.

In the 1^st^ phase, the “exploratory”, there was a meeting between researchers and nurses from the Technical Chamber of Obstetric Nursing, in which an attempt was made to diagnose weaknesses in the preliminary instrument’s structure and to identify ways to assess validity evidence.

In the 2^nd^ phase, called “the research theme”, the problem and the area of knowledge were designated. In this phase, the researchers deepened their studies in search of the theoretical framework and adopted psychometrics, as proposed by Pasquali^(16)^, as a frame of framework. At that moment, two psychometrists were invited to participate as researchers in the project to collaborate in instrument assessment and improvement.

In the 3^rd^ phase, designated as “posing the problem”, developed between January and March 2020, it aimed to analyze the preliminary instrument’s structure and internal consistency. For this, the results of the application of this instrument in a public hospital in FD were analyzed.

This preliminary instrument consisted of 15 items, structured by objective questions with a scale of dichotomous and polytomous responses. The instruments had already been applied in a pilot test. Their results were filed in folders at the health service itself. Data collection was through the unarchiving of these instruments. Those filled out in more than 50% of items were included. Those with more than 50% of non-completion were excluded. Then, data tabulation and exploratory factor analysis were performed using the Statistical Package for the Social Sciences (SPSS), version 26, and Factor Analysis. The results supported the problem verification and demarcation as well as the design of proposed solutions.

In the 4^th^ phase, known as “the place of theory”, a literature review was carried out to better understand the object of study and map the validated instruments used to assess women’s satisfaction with the obstetric care received during labor and childbirth.

A search was carried out in the PubMed, Virtual Health Library (VHL), Cochrane and SciELO databases, using the indexed descriptors “Patient Satisfaction”, “Childbirth” and “Validation Studies”, applying Boolean operators and filters. This phase was concentrated between September and December 2019 and, subsequently, this literature review was structured in a scope review article “Childbirth care service assessment: a scoping review of measurement instruments”, submitted in a journal scientific.

In the 5^th^ phase, called “hypotheses”, the researchers compared the preliminary instrument with the validated instruments identified in the previous phase. Hypotheses and proposals were prepared to improve the preliminary instrument, listing the main weaknesses and needs for adjustments.

The 6^th^ phase, designated as “seminar”, was carried out with nurses from the Technical Chamber of Obstetric Nursing, through a remote focus group, between March and May 2020. The researchers presented the results of the 3^rd^, 4^th^ and 5^th^ phases to nurses, with the purpose of discussing and making decisions regarding the need to improve the preliminary instrument. At that moment, reflection was encouraged based on the weaknesses identified in the analysis of the preliminary instrument’s structure and literature review result synthesis. Then, the discussion of the proposals, elaborated in the 5^th^ phase, was encouraged, to carry out construct improvement. The focus group technique was adopted, in which group interviews are configured, through data collection through interactions that occur between those involved^(17)^.

Nurse-midwives from the Technical Chamber of Obstetric Nursing who were available to participate on the agreed days and times were included. Nurses who were away from activities in the technical chamber due to legal leave were excluded. Data were collected through two previously scheduled and recorded remote meetings. Seven days before the meetings, participants received the instrument to be improved so that they had enough time to analyze it. The first lasted 90 minutes, and the second, 40 minutes. Participants agreed to participate by signing the Informed Consent Term (ICF). In this phase, the COnsolidated criteria for REporting Qualitative research (COREQ) checklist was used for focus groups^(18)^.

Data analysis occurred through transcription of speeches, reading, rereading and review of textual description. Subsequently, the main meanings were identified in each part of the body of the text that led to the group’s consensus in making changes to each preliminary instrument’s item, which generated a more improved version of the instrument.

The 7^th^ phase, called “field of observation, sampling and representativeness”, took place together with the 8^th^ phase “data collection”. They took place between May and October 2021, and aimed to assess the target audience’s understanding of the instrument content and clarity being improved, in addition to assessing the perception of satisfaction with childbirth care. Postpartum women who were admitted to a RI of a public hospital in FD in two specific beds were selected as a way to minimize selection bias. Data were collected through individualized and semi-structured interviews, containing open-ended questions.

Mothers in good health were included, who had all childbirth care in this service, where the result of childbirth was a live birth and who stayed at least 24 hours hospitalized in the health unit. Underage mothers who had some serious mental or physical disability that made it impossible to speak or move to the reserved room where the interview took place were excluded. All participants signed the ICF. In this phase, the COREQ checklist criteria were used for interviews^(18)^.

At the beginning of the interview, the instrument being improved was presented, asking users to assess it by reading and rereading it completely without answering it. After some time, the researcher would return to the room, bringing the following guiding question: by analyzing the instrument, is there any term of the satisfaction assessment instrument that you did not understand? The other questions addressed the assessment of satisfaction with the childbirth care received.

The interviews were recorded and transcribed. Then, the text was revised and, subsequently, the data were prepared for analysis in *the Interface de R pour les Analyzes Multidimensionnelles de Textes et de Questionnaires* (IRAMUTEQ)^(19)^.

In the 9^th^ phase, which is configured as “learning”, there was a moment of grouping the information, assessment and synthesis to establish the necessary changes in the construct restructuring based on literature review and existing statistical measures.

Then proceeded to the 10^th^ phase, known as “formal knowledge/informal knowledge”. In this phase, “formal knowledge” coping from the contributions of nurse-midwives participating in the focus group and synthesis produced by the researchers in the 9^th^ phase with the “informal knowledge” derived from the interviews with puerperal women to guide the final structuring of the instrument.

In the 11^th^ phase, designated as the “action plan”, there was a convergence of what was produced in all the previous phases to establish a consensus on the results and conclude with the improved version of the Instrument for Assessing Postpartum Women’s Satisfaction with Childbirth Care.

In the 12^th^ second phase, named “dissemination of results”, the improved version of the instrument was delivered to the Technical Chamber of Obstetric Nursing.

## RESULTS

Following the designs carried out in the 1^st^ and 2^nd^ phases, in the 3^rd^ phase, 372 instruments of the preliminary version applied were analyzed. Initially, the Kaiser-Meyer-Olkin (KMO) criterion was 0.837, and Bartlett’s sphericity test was statistically significant (p<0.001), demonstrating that the data could be submitted to exploratory factor analysis^(16)^. Then, data were transformed into a Z score, as they presented different response scales, and the parallel analysis suggested the existence of three factors for assessing the construct (Chart 1).

**Chart 1:** Result of the exploratory factor analysis of the preliminary version of the instrument for assessing postpartum women’s satisfaction with childbirth care, Brasília, Distrito Federal, Brazil, 2020

	1	2	3
**12**- In the maternity ward, what is your level of satisfaction with the guidelines given by the nursing team on postpartum care?	0.773		
**14-**In general, what is your level of satisfaction with the care you received in the obstetric services of this unit?	0.67		-0.421
**11-** In the maternity ward, what is your level of satisfaction with the guidelines given by the nursing team regarding care for the baby (cleaning the stump, burping position, bathing?)	0.664		
**13-** In the maternity ward, what is your level of satisfaction with the guidelines given by the Milk Bank on breastfeeding?	0.608		
**01-** On the day of childbirth, how was the service at the hospital reception?	0.391		
**15-**Your experience of labor, childbirth and postpartum	0.337		
**03-**Assess the quality of care received in risk stratification	-	-	-
**06-**What was your type of childbirth	-	-	-
**09-** How often did the nursing staff explain things to you in a way that you could understand?		0.879	
**10-** How often did the medical team explain things to you in a way that you could understand?		0.411	0.318
**04-** Was the companion of your choice allowed to enter the Obstetric Center during labor, childbirth and postpartum?		-0.367	
**02-**Did you go through screening and risk stratification (did you wear a bracelet)?	-	-	-
**08-** How often did the medical team treat you with courtesy and respect?			0.731
**07-** How often did the nursing staff treat you with courtesy and respect?			0.64
**05-** How often do health professionals introduce themselves by name and role?			0.34

In the three-factor structure, there was the presence of mixed items with loads greater than 0.30 in more than one factor (10 and 14), the loss of three items from the instrument (2, 3 and 6) and the grouping of items into factors with different content than expected. As an example, the constant items in factor 1 assessed satisfaction, although two items changed the meaning for childbirth experience and experience with care. Thus, the three factors did not appear to be theoretically consistent. Moreover, Cronbach’s alpha was 0.468, a value considered unacceptable^(20)^. The root mean square error of approximation (RMSEA) = 0.048, goodness of fit index (GFI) = 0.901 and Tucker-Lewis index (TLI) = 0.915 were adequate, but the comparative fit index (CFI) = 0.949 was not adequate^(20)^. From this, instrument modifications were recommended.

In the 4^th^ phase, in which the literature review took place, 16 published articles were identified, describing validity studies on satisfaction with obstetric care during childbirth. Among these, the analysis was deepened, in particular, in three instruments validated in Brazil: Childbirth Experience Questionnaire^(21)^; Mackey Childbirth Satisfaction Rating Scale^(22)^; and Birth Satisfaction Scale-Revised^(23)^. These instruments were used in several studies in different countries^(24-26)^, including Brazil.

Considering the results of the 3^rd^ and 4^th^ phases, in the 5^th^ phase, “hypotheses” were proposed, a greater investment in theoretical aspects and coverage on construct assessment. It was also indicated that other properties of the instrument should be adjusted, such as the response scale standardization, item selection and organization, scale intensity degree and content reformulation.

Then, in the 6^th^ phase, “seminar”, participants were presented with the result of the instrument’s factor analysis and the data of analyzed articles that measured women’s experience in childbirth, with the aim of demonstrating suggestions for themes that could be included in the instrument. The particularities and differences in each instrument were highlighted, so that the participants could have a more critical view of content. Then, group discussions followed the guiding questions: what contents and constructs could be inserted into the instrument? What modifications are needed to the current instrument?

Nurse-midwives participated by suggesting, questioning and collectively contributing to this reflective process to improve the instrument. Each item was discussed and, finally, when necessary, the structure of the new variable was written, making item wording clear as a result of participants’ consensus. All these interactions made it possible to explore the various characteristics that assess hospital childbirth and allowed for a more critical examination of instrument improvement. Modifications to the instrument were described in Chart 2.

**Chart 2:** Items maintained, created and modified from the preliminary version of the instrument by nurse-midwives during a focus group, March to May 2020, Brasília, Distrito Federal, Brazil

MAINTAINED ITEMS	CREATED ITEMS	MODIFIED ITEMS
*01- On the day of childbirth, how was the service at the hospital reception?*	*06-You were given the opportunity to participate in decisions and procedures during work. How do you rate?*	*02-Assess the quality of care received in the risk classification or triage (Room where the nurse places a colored bracelet on your arm).*
*04- Was the companion of your choice allowed to enter the Obstetric Center during labor, childbirth and postpartum?*	*07-How do you rate the hospital environment where your childbirth took place.*	*03-How do you rate your partner’s collaboration?*
*05- How often do health professionals introduce themselves by name and role?*	*08-How do you rate respect for your body during childbirth. Example: The moment professionals examine your body.*	*09-How do you rate the guidelines given by the medical team during childbirth.*
*11- In the maternity ward, what is your level of satisfaction with the guidelines given by the nursing team regarding care for the baby (cleaning the stump, burping position, bathing?)*		*10-How do you rate the nursing team’s guidelines during childbirth.*
*13- In the maternity ward, what is your level of satisfaction with the guidelines given by the Milk Bank on breastfeeding?*		*12-In the maternity ward, what is your level of satisfaction with the guidelines given by the health team on breastfeeding*

Finally, in the second meeting, they defined a four-point Likert-type scale (very satisfied, satisfied, dissatisfied and very dissatisfied) as predominant in most of the instrument and approved the new version, which was composed of 13 items, in which the conceptual framework has become more focused on assessing user satisfaction. Items were organized from more general to more specific contents, and a four-point Likert-type scale with inverted degree of intensity was standardized.

Proceeding to the 7^th^ and 8^th^ phases, 20 puerperal women aged between 18 and 35 years old participated, 50% of whom were primiparous, 35%, second parities, and 15%, multiparous. Regarding education, 16 interviewees had completed high school or higher education level.

**Figure 1:**
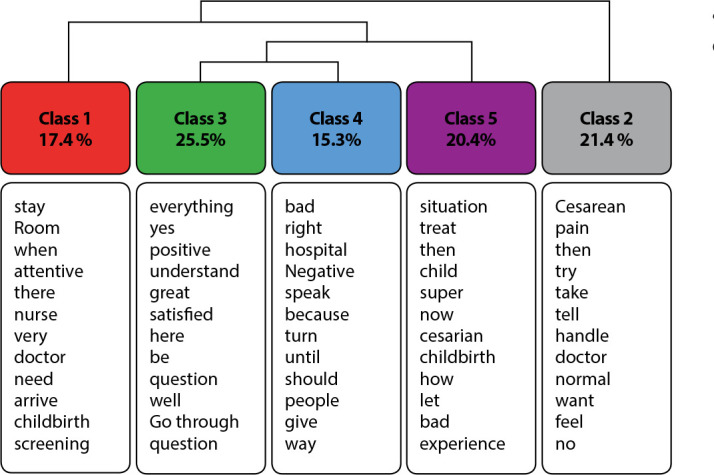
Dendrogram referring to the distribution of the vocabulary of the classes according to the Descending Hierarchical Classification in relation to satisfaction with childbirth care, Brasília, Distrito Federal, Brazil, 2021

The general corpus consisted of 20 texts, separated by 146 text segments (TS), with 98 TS used (67.12%) of the total of 146 TS. A total of 4,819 occurrences emerged (words, forms and vocabulary). The corpus generated a main class (2), which was subdivided, giving rise to class 1. From the branching of the previous step, there was a subdivision of the branch, originating class 5, where it branched, generating classes 3 and 4 (Figure 1).

The thematic categories were described below:

### Individualized care

This class has 17 TS, corresponding to 17.4% of the total analyzed corpus. Humanized care that encourages the role of women during childbirth proved to be significant in the perception of puerperal women’s satisfaction, perceived in the following statements:

*The girls who stayed with me there in the pre-childbirth period, wow, there was immense attention on us the whole time, very attentive and affectionate, I liked it a lot.* (Desert Flower)

*Right at the time of childbirth, while I was powerless to finish pushing the baby, the nurses who were on duty encouraged me positively.* (Iris)

### The choice of childbirth route

This class contains 21 TS, representing 21.4% of the corpus. The mode of childbirth proved to be an important criterion in assessing women’s satisfaction during childbirth, as this form of birth interferes with the various psychobiological aspects of parturient women, as described in speeches:

*Because I have thrombocytopenia, they wanted to have a normal childbirth so as not to lose so much blood, but then they tried to induce, trying to induce, feeling a lot of pain, but then it wasn’t dilating, so they had to go to the cesarean section.* (Gardenia)

*Look, I’m not going to lie to you, the first consultation was horrible, the doctor didn’t cooperate with all the paperwork I brought because I was from outside and had brought a report that had to be a cesarean section and she forced it to be normal, I was very dissatisfied.* (Azaleia)

### Instrument assessment

This class comprises 25 TS, featuring 25.5% of the corpus. The instruments for assessing puerperal women’s satisfaction were identified as necessary for the health system, as they serve as a mechanism to listen to the population and improve services, as reported in speeches:

*There should be this survey in all hospitals about care, as you went through, it would be really cool for people to go through this.* (Lily)

*This questionnaire is necessary because, sometimes, people are treated badly and do not have the right to open their mouths and speak, if everyone were like this, a lot would change, if all places were like this, there was the opportunity to speak, then it would be better.* (Amarylis)

### Communication

This class has 15 TS, representing 15.3%. Communication is relevant in the assessment of postpartum women’s satisfaction, demonstrating the importance of improving the dialogue between user and health professional, as shown below:

*I think they should listen a little more. And they don’t give us a voice, just medical protocol history and they ended up forgetting me* [...] *then we end up living a bad experience because of that, because they don’t give us the right to make our choices* [...]. (Iris)

*Here, just for lack of information, some are very helpful, others come in and don’t give you information, they don’t give you all the information you need, others don’t even know how to answer your questions, they don’t know how to explain what’s going on.* (Melissa)

### Treatment perception

This class has 20 TS, corresponding to 20.4%. Pregnant women’s expectations and what actually happens during childbirth revealed that such aspects can substantially interfere with puerperal women’s perception of satisfaction, as pointed out in the testimonies:

*According to the comments from people outside, I was very scared of having come here to have my son, but, incredible as it seems, I was very well assisted.* (Desert Flower)

*In my case, what happened is that they were only taking into account what was best for my daughter and ended up forgetting about me, and that was what ended up aggravating the situation and leaving me like this, really down.* (Iris)

Finally, the 9^th^ to 12^th^ phases were carried out, in which the new version of the improved instrument was constructed and delivered to the Technical Chamber of Obstetric Nursing.

## DISCUSSION

The Instrument for Assessing Postpartum Women’s Satisfaction with Childbirth Care improvement was the result of an action-research process consisting of twelve phases, in which, through a joint action of the various participants, they promoted the improvement of this construct as a tool for maternal and child health management.

The literature review supported greater theoretical anchoring for study participants, which helped reflection and discussion on the instrument’s content. It reinforced similar aspects found in the validated instruments as well as the identification of gaps and weaknesses in the preliminary instrument.

In the exploratory factor analysis of the preliminary instrument, a lack of standardization of the instrument’s response scales was identified. Several authors indicate that when the scale has between four and six response options, it has better psychometric properties^(27-28)^. Thus, given the flaws in the preliminary instrument’s structure, there was a need to review its structure in order to improve it and make it more reliable to measure satisfaction with childbirth care.

The stage performed with nurse-midwives was configured as a moment of social participation that contributed to the process of improving the instrument. All dynamics occurred jointly between the researcher and participants, seeking to maintain actors’ involvement in the process^(15)^. Experts’ opinion and clinical observation are significant tools in the stages of constructing the items and defining the instrument’s domains^(29)^. Therefore, the meeting with the creators of the preliminary instrument allowed the analysis of the instrument’s context and its structure.

The preliminary instrument had contents such as pregnant women’s participation in childbirth, multidisciplinary assessment, guidance, newborn care, ambience and companion role. These are components of a basic structure present in most validated instruments that assess postpartum women’s satisfaction^(30)^. One of the most relevant steps in the construction of an instrument is the operationalization of the constructs to be assessed^(16)^. These are key steps in the construction of health measurement instruments, highlighting the specific operational definitions of the construct as elementary, which directly influence validity evidence^(29)^.

Puerperal women collaborated for item construction and adjustments, according to the report of their experiences^(31)^. Among the most discussed topics, the lack of dialogue with professionals emerged as well as in other studies^(32)^. Therefore, clear and timely communication generates confidence in the parturient, contributing to labor’s good evolution^(33)^. Every pregnant woman has the right to information, respect for their feelings as well as explanations about the obstetric procedures taken, thus maintaining good dialogue and strengthening a more humanized childbirth^(11)^.

Humanized care during childbirth was also highlighted as essential by mothers. For this, assistance must be guided by respect, safety, comfort, privacy, good dialogue, maintaining a calm and peaceful environment, in addition to other attitudes that will contribute to the smooth running of childbirth^(34-36)^ and, consequently, to puerperal women’s satisfaction.

On the other hand, dissatisfaction with obstetric care is mainly related to the units’ infrastructure and the relationship between user and health team^(31-32)^. According to the reports of puerperal women in classes 4 and 5, some health professionals systematically follow protocols or institutional routines. They consider themselves holders of knowledge, treat users as mere executors of guidelines and fail to provide holistic care, meeting parturient women’s real needs^(37)^. Therefore, despite the high rate of satisfaction with childbirth care^(38-40)^, data related to dissatisfaction reveal the need for constant investment in strategies to solve these problems inherent in childbirth care.

During puerperal women’s speeches, another emerging theme was childbirth route. Brazil has a high cesarean section rate, around 40% in public services, despite the fact that most women assisted in these services have a preference for vaginal childbirth^(41-42)^. Knowing parturient women’s opinion about what they expect from childbirth is important, as it will imply their level of satisfaction at the end of the process, since often their childbirth experience does not meet their expectations^(43)^. The choice or preference for childbirth route, when not very well clarified and shared, generates conflicts in users^(44)^. Thus, there is a need to explain to pregnant women about the risks and benefits of each type of childbirth, respecting scientific evidence and women’s autonomy so that they can decide, when possible, together, the best childbirth route^(45)^. Therefore, it is understood that the best way in childbirth planning to minimize traumas and frustrations is linked to an open dialogue, implementation of good childbirth practices and multidisciplinary support.

Puerperal women understood that the instrument is an opportunity to express their opinion about the care received, and they had no difficulty in interpreting the items and saw it as a means of being heard in the public health service. This assessment stage of the instrument being improved by the target population contributed positively to an important phase in the instrument validation process, which corresponds to semantic validity^(16)^. Thus, these postpartum satisfaction assessment surveys strengthen this result indicator as a management tool, corroborating with community participation and for health service assessment, providing better quality assistance to women^(46-47)^.

### Study limitations

The COVID-19 pandemic limited the execution of focus groups in virtual environments. If they had taken place in person, they could have generated other perspectives and different contributions.

### Contributions to nursing, health, or public policies

The use of action research as a method made it possible to contribute scientifically to improving a useful tool to assess postpartum women’s satisfaction in relation to the childbirth experience. The participation of the actors involved in the instrument elaboration ensured that the expectations of when the instrument was created were met, in addition to maintaining their involvement with it, favoring greater social insertion.

## FINAL CONSIDERATIONS

Improving the Instrument for Assessing Postpartum Women’s Satisfaction with Childbirth Care was a laborious process, but it was observed that each phase of the action research in this study potentially contributed to its improvement, resulting in a more appropriate and accurate instrument. The involvement of nurse-midwives who prepared the preliminary version of the instrument encouraged greater commitment to the use of this tool in obstetric services, subsequently.

Considering that health measurement instruments are relevant elements for clinical practice and health policies, future research is needed to assess the evidence of validity and reliability of this improved instrument.
